# Clinical Features and Outcomes Analysis of Surgical Resected Pulmonary Large-Cell Neuroendocrine Carcinoma With Adjuvant Chemotherapy

**DOI:** 10.3389/fonc.2020.556194

**Published:** 2020-12-01

**Authors:** Yinchen Shen, Fang Hu, Changhui Li, Jianlin Xu, Runbo Zhong, Xueyan Zhang, Tianqing Chu, Baohui Han

**Affiliations:** Department of Pulmonary Medicine, Shanghai Chest Hospital, Shanghai Jiao Tong University, Shanghai, China

**Keywords:** large-cell neuroendocrine carcinoma, adjuvant chemotherapy, prognosis analysis, driven genes, serum tumor markers

## Abstract

**Objective:**

Large-cell neuroendocrine carcinoma (LCNEC) is a rare subtype of pulmonary cancer with poor survival. Optimal adjuvant chemotherapy for resected LCNEC is controversial till now; clinical features together with the prognostic factors in LCNEC should be clarified better.

**Methods:**

Clinicopathological characteristics, driven genes’ status (EGFR, ALK, and ROS1), adjuvant chemotherapy strategy for 94 surgical resected LCNECs were extracted from digital database, tumor relapse or progression, and survival were analyzed with clinical profiles.

**Results:**

Driven gene mutants were scarce in LCNEC, 8.3% (4/48) samples harbored EGFR mutations, 5.8% (3/52) with ALK positive, and none of ROS1 positive. A total of 44 patients suffered tumor relapse or progression during follow-up. Tumor/lymph node (N) stage, serum carcinoembryonic antigen (CEA) level before surgery, different adjuvant chemotherapies were associated with tumor relapse (*P* < 0.05); poorer disease-free survival (DFS) appeared in N2/stage III, serum CEA positive and pemetrexed based chemotherapy (*P* < 0.05); for overall survival (OS) analysis, the T/tumor stage, serum positive CEA/neuron-specific enolase (NSE) at baseline were associated with worse OS (*P* < 0.05). Moreover, in the multivariate analysis, N stage still acted as prognostic for DFS (*P* = 0.019); OS differed significantly in different T stages, chemotherapy selection and serum CEA levels after adjustment (*P* < 0.05).

**Conclusion:**

Classical driven gene mutations were rare in LCNEC. Tumor N stage appeared as prognostic for DFS, while serum positive CEA, different adjuvant chemotherapy strategies, and T stage were independent prognostic factors for OS. Etoposide–platinum regime seemed to be a better choice which should be confirmed by further prospective investigations.

## Introduction

Large-cell neuroendocrine carcinoma (LCNEC) is a rare type of lung cancer, which accounts lower than 3.5% of all (1, 2), while according to the Surveillance, Epidemiology, and End Results (SEER) (2001–2007) database, LCNEC incidence seemed to increase (3). Since this subtype is a high-grade malignancy and presents as neuroendocrine features (4), LCNEC is used to be a subcluster of large-cell carcinoma (LCC) and part of neuroendocrine tumors (NETs) of the lung before 2015, and the World Health Organization (WHO) lung tumor classification revised the criteria (2015) which moved LCNEC from LCC to NET chapter (5). Previous reports indicated LCNEC appeared aggressive and the prognosis was poor (6, 7) and shared some similarities with small-cell lung cancer (SCLC) (8) or non-small cell lung cancer at the same time (9).

The rarity of LCNEC impeded large-scale randomized clinical trials in seeking the optimal therapy; majority of the present were data derived from retrospective studies, and the sample size was also small. Similar to NSCLC, early stage (stages I–II) LCNEC usually received surgical resection, while for local advanced or metastatic tumors, the treatment selection is still controversial, either for adjuvant chemotherapy or first-line therapy. Reported data evaluated platinum–etoposide combination, which was widely used in treating SCLC, as a better choice for prolonging survival (1, 10, 11); however, most of the results focused on IIIB/IV stage tumors, and treatment for patients with operation should be clarified further.

As targeted therapy provided a promising prognosis for specific patients in NSCLC, driven gene detection is necessary before clinical decision, while gene mutant data related to LCNEC at present was rare. Recently, Zhuo et al. reported genetic subtyping was associated with tumor prognosis (12), which indicated treatment selection might rely on genomic status. Considering the gloomy outcomes in LCNEC, clinical characteristics, genomic information, and survival should be investigated with deeper insight. Herein we conducted this retrospective study to provide an overview of LCNEC in Chinese population, especially for resected tumors; the adjuvant chemotherapy effects, driven gene spectrum and survival will be concentrated in order to help understand LCNEC better.

## Materials and Methods

### Study Population

During August 2011 to October 2019, a total of 105 LCNEC underwent surgical resection in Shanghai Chest Hospital, and all samples were confirmed as LCNEC or combined LCNEC (N = 11) following the 2015 WHO lung tumor classification criteria (13), and only LCNECs were collected (N = 94). Informed consent was obtained from all patients, and the present study was approved by the Institutional Review Board (IRB) in ShangHai Chest Hospital [No. KS(Y)1982].

### Data Extraction

An independent database was established based on hospital digital medical records; details of these individuals were extracted such as patients’ age, gender, smoking status, primary tumor size, tumor location, tumor-nodal-metastasis (TNM) staging information, peripheral blood tumor marker carcinoembryonic antigen (CEA), squamous cell carcinoma antigen (SCC), cytokeratin-19 fragment (CYFRA21-1), neuron-specific enolase (NSE), cancer antigen-125 (CA125), and gene detection results. Blood tumor markers were evaluated before surgery, epidermal growth factor receptor (EGFR) mutants were detected with amplification refractory mutation system (ARMS), anaplastic lymphoma kinase (ALK) rearrangement was detected by immunohistochemistry (IHC), and ROS1 fusion was determined with fluorescence *in situ* hybridization (FISH). All tumor stage was performed according to the Eighth edition of American Joint Committee on Cancer (AJCC) staging system (14).

### Patient Follow-Up and Definition of End-Point

Serial clinical physical examination and image evaluation (included chest computed tomography, brain magnetic resonance imaging, abdomen ultrasound or whole-body^18^ F-Fluorodeoxyglucose positron emission tomography/computed tomography if necessary) were recommended to all patients in a sequential follow-up. Disease-free survival (DFS) was defined as the time from surgery to the first confirmed relapse or alive at final follow-up; overall survival (OS) was defined as time to death by any cause or last follow-up from diagnosis. Survival information was collected mainly by phone communication and outpatient visit. Last follow-up date was set at November 2019.

### Statistical Analysis

The Chi-square (χ2) test or Fisher’s exact test was used for clinicopathological characteristics comparison analysis in LCNEC. Survival differences were analyzed by Kaplan–Meier survival function with the method of log-rank test. Moreover, variants including age, gender, tumor location and size, tumor staging, chemotherapy or radiotherapy status, and peripheral blood tumor markers were evaluated by fitting logistic multivariable regression with Cox proportional hazard models. All statistical analyses were performed by the SPSS 19.0 statistical software (SPSS, Inc., Chicago, IL, USA) and GraphPad Prism 7 (GraphPad Software, Inc.); significance level was set at two-sides *P* < 0.05.

## Results

### Characteristics of Resected LCNEC Patients

Among the 94 LCNEC patients, 84 (89.4%) were males, and 10 (10.6%) were females; median age was 60 (range 35–80 years) and 35 (37.2%) were current or former smokers. More than half (60, 63.8%) of the patients had a tumor with diameter larger than 3 cm, and 64 (68.1%) patients had tumors located in the left lobe. Stage I, II, and III tumors accounted for 33% (31/94), 23.4% (22/94), and 43.6% (41/94), respectively. Of the 94 patients, three received neoadjuvant chemotherapy, and all these three patients refused the adjuvant chemotherapy after surgery. 75 (79.8%) patients received adjuvant chemotherapy, of which pemetrexed/cisplatin (PEM) or carboplatin contained 26 (34.7%), and etoposide–platinum (PE) based regime contained 21 (28%), 28 (37.3%) were gemcitabine/vinorelbine/paclitaxel–platinum (GVTP). A total of 38 patients received radiotherapy of which 16 (42.1%) were followed by chemotherapy, and 22 (57.9%) received radiotherapy for tumor recurrence. Detailed clinicopathological characteristics of the study patients were presented in **Table 1**.

**Table 1:** Clinicopathological characteristics of 94 resected LCNEC patients.

Characteristics	Number (%)
Gender	
Male	84 (89.4)
Female	10 (10.6)
Age	
≥60	55 (58.5)
<60	39 (41.5)
Smoking history	
Yes	35 (37.2)
No	52 (55.3)
Missing	7 (7.5)
Primary tumor location	
Left upper	30 (31.9)
Left lower	11 (11.7)
Right upper	27 (28.7)
Right lower/middle	26 (27.7)
Tumor size (cm)	
>3	60 (63.8)
≤3	34 (36.2)
Tumor stage	
I	31 (33.0)
II	22 (23.4)
III	41 (43.6)
Adjuvant chemotherapy strategy	
PEM	26 (34.7)
PE	21 (28.0)
GVTP	28 (37.3)
CEA (ng/ml)	
Positive (>5)	23 (27.4)
Negative (≤5)	61 (72.6)
SCC (ng/ml)	
Positive (>1.5)	9 (10.7)
Negative (≤1.5)	75 (89.3)
CYFRA21-1(ng/ml)	
Positive (>5)	12 (14.3)
Negative (≤5)	72 (85.7)
NSE (ng/ml)	
Positive (>25)	13 (15.5)
Negative (≤25)	71 (84.5)
CA125 (U/ml)	
Positive (>35)	11 (13.1)
Negative (≤35)	73 (86.9)

### Serum Tumor Biomarkers Level and Genetic Alternations Profiles of LCNEC

Five kinds of peripheral blood tumor biomarkers were selected for evaluation, which included CEA, SCC, CYFRA21-1, NSE, and CA125. Positive rates of these biomarkers were 27.4% (23/84) for CEA, 10.7% (9/84) for SCC, 14.3% (12/84) for CYFRA21-1, 15.5% (13/84) for NSE, and 13.1 (11/84) for CA125. Furthermore, common driven genes such as EGFR, ALK, and ROS1 mutations were confirmed in this cohort; mutant status was available in 51.1% (48/94), 55.3% (52/94) and 26.6% (25/94), respectively. Four (8.33%) patients harbored EGFR mutations, of which two were L858R and two with 19 deletions. 5.77% (3/52) of patients appeared ALK positive, and all ROS1 status was negative in the present study.

### Outcomes Predictors With Univariate Analysis in LCNEC

**Figure 1:**
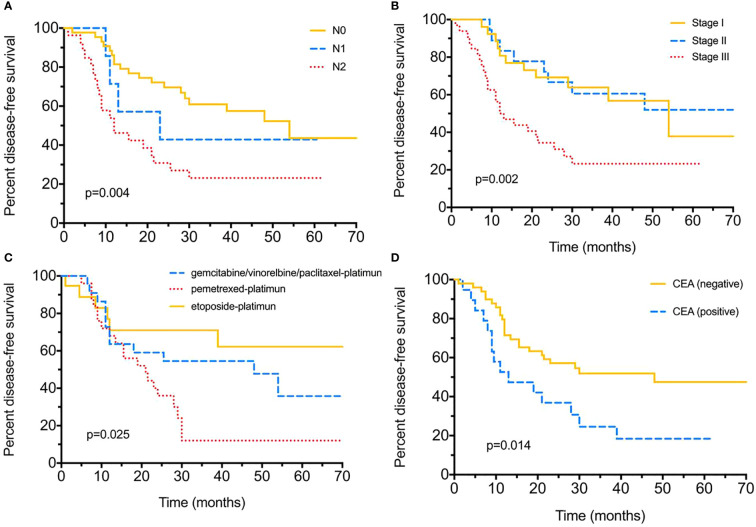
Disease-free survival (DFS) for surgical resected LCNEC. **(A)** Disease-free survival by different nodal (N) stages. **(B)** Disease-free survival by different tumor stages. **(C)** Disease-free survival with different adjuvant chemotherapy strategies. **(D)** Disease-free survival between positive/negative serum CEA levels.

**Figure 2:**
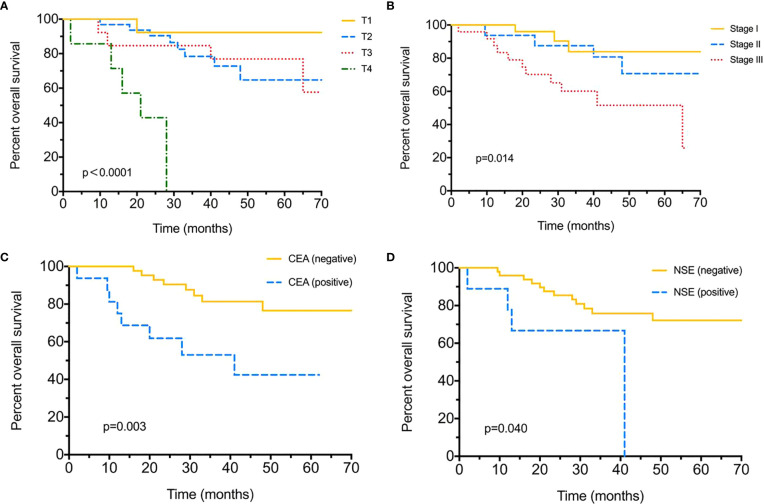
Over-all survival (OS) for surgical resected LCNEC. **(A)** Over-all survival by different T (tumor) stages. **(B)** Over-all survival by different tumor stages. **(C)** Over-all survival between positive/negative serum CEA levels. **(D)** Over-all survival between positive/negative serum NSE levels.

Until the final follow-up, we obtained information of tumor relapse in 84.04% (79/94) of patients, and 55.7% (44/79) suffered relapse or tumor progression during the follow-up, of which nine (20.5%) had intrapulmonary tumor recurrence, eight (18.2%) with brain and five (11.4%) with bone metastasis, eight (18.2%) suffered lymph node metastasis, and nine (20.5%) already died (**Table 2**). The tumor/nodal (N) stage was significantly associated with recurrence, with *P =* 0.021 and 0.022. Positive serum CEA level (>5 ng/ml) appeared to be more likely with relapse (75% *vs* 49%, *P =* 0.047); moreover, different chemotherapies were also associated with tumor recurrence (*P* < 0.005). As for DFS evaluation, N2 tumor indicated poorer DFS (median 54-N0 *vs* 23-N1 *vs* 12-N2 months*, P =* 0.004), and tumor stage (median 12 months in stage III), pemetrexed–platinum based chemotherapy (median 21 months) and serum CEA positive were also significantly with worse DFS (median 48-positive *vs* 13-negtive months), with *P =* 0.002, *P =* 0.025, *P =* 0.014, respectively (**Figure 1**). Furthermore, median DFS was longer with PE than with others (not reached), and PEM indicated the worst survival (21 months). Over-all survival was analyzed in 77.66% (73/94) of patients. T (tumor) stage (*P* < 0.0001), tumor stage (*P =* 0.014) and serum positive CEA (*P =* 0.003)/NSE (*P =* 0.04) at baseline were all significantly associated with shorter OS (**Figure 2**); furthermore, different chemotherapy regimes also appeared a significant trend (*P =* 0.059) (**Supplement Figure 1**).

**Table 2:** Tumor relapse/progression patterns in 44 surgical resection LCNEC patients.

Relapse/progression patterns	Number (%)
Intrapulmonary	9 (20.5)
Brain metastasis	8 (18.2)
Bone metastasis	5 (11.4)
Lymph node metastasis	8 (18.2)
Death	9 (20.5)
Abdomen metastasis	
Liver	2 (4.5)
Pancreas	1 (2.2)
Adrenal gland	1 (2.2)
Abdominal cavity	1 (2.2)

### Multivariate Analysis of Outcomes Predictors in LCNEC

In the multivariate analysis, T and N stages, peripheral blood CEA/NSE level, tumor stage and chemotherapy in relation to patients’ DFS and OS were selected. The N stage still acted as an independent prognostic factor for DFS (*P =* 0.019), and OS differed significantly in different chemotherapies (*P =* 0.027), T stage (*P =* 0.01), and serum CEA levels (*P =* 0.032) after adjustment. No other associations were discovered in survival analysis (**Table 3**).

**Table 3:** Multivariate analysis of outcomes predictors in LCNEC patients.

Prognostic characteristics	P value	DFS HR	95% CI	P value	OS HR	95% CI
T stage	0.15	1.51	0.86–2.66	0.01	2.39	1.23–4.63
N stage	0.019	1.53	1.07–2.19	0.11	3.26	0.76–13.96
TNM stage	0.26	0.53	0.17–1.61	0.12	0.22	0.035–1.45
Chemotherapy	0.43	0.81	0.47–1.38	0.027	0.30	0.10–0.87
CEA	0.28	1.58	0.69–3.61	0.032	4.20	1.14–15.49
NSE	0.77	1.19	0.37–3.78	0.60	1.61	0.27–9.62

## Discussion

Since LCNEC was a rare type of lung cancer, the reported results were scarce and mostly derived from small sample size studies. Moreover, comprehensive analysis was limited, and clinical management for LCNEC remains controversial in some respects (10, 12, 15, 16). Hence we conducted this retrospective study in order to give an overview the clinical characteristics and prognostic variants of LCNEC. Diagnosis and treatment of lung carcinomas thrived dramatically, while few data was related to uncommon cancer types. We provide the landscape of tumor relapse and adjuvant therapy for resected LCNEC and confirmed PE was a priority for these patients; furthermore, normal serum tumor markers such as CEA and NSE could be utilized for prognosis evaluation, which was convenient and non-invasive for clinical practice.

Like most reported studies, LCNEC more likely occurred in males, with 89.4% in our study and 62.5–90.6% in others (2, 10, 12, 15, 16). Driven genes such as EGFR and ALK forecasted targeted therapy in NSCLC and detected as routine in clinical management. As for LCNEC, we found EGFR and ALK mutants were both rare in this subtype; the mutation rates were 8.33 and 5.77% respectively. Naidoo et al. also evaluated these genes in 49 LCNECs, they discovered none EGFR mutation or ALK rearrangement in 17 patients (15); however, 24% (4/17) harbored KRAS mutants. Considering all recruited patients were stage IV, the genomic alternations might differ between different tumor stages, which should be investigated in the future, and whether targeted therapy could be used was also controversial.

CEA is a widely used serum tumor marker and if positive before surgery seemed to be a risk in tumor relapse; moreover, positive CEA is also significantly associated with poorer DFS and OS in LCNEC. Zhang et al. also evaluated CEA in LCNEC prognosis, and no significance was mentioned (10), while 30.7% (117/381) in the study were stage IV patients. The mixed groups induced different proportions of positive CEA in the whole cohort, for 27.4% in our study and 42.2% in theirs (n = 301). Metastasis always obtained heavy tumor burden, which influenced the CEA level in the peripheral blood. Kim et al. collected 139 LCNEC patients who received operation; however, no tumor marker information was involved (16). Positive NSE at baseline was significantly associated with shorter OS in the univariate analysis although only 15.5% was positive in the present study, and 50.6% (n = 241) in Zhang et al.’s (10). The result was also consistent with theirs. However, 36.7% of the samples lacked the NSE information, and tumors involved in the final analysis would be different between studies since NSE was specific for NETs. Maybe further investigation could notice this.

Due to lack of randomized clinical trials in adjuvant treatment for LCNECs, the optimal therapy in these patients was still in debate. In a large scale investigation whether adjuvant treatment could benefit LCNECs, patients with stage II or higher seemed to obtain better DFS and OS (16); however, the chemotherapy information was not provided. Although some previous researches evaluated different treatments in LCNECs, Treut et al. demonstrated that cisplatin–etoposide doublet may induce poor survival with advanced LCNEC (11). Another study chose platinum–etoposide in metastatic LCNEC, with 37% objective response rate (ORR; complete response + partial response) (15), and no response to other regimens. Metro G et al. investigated the survival outcomes and incidence of brain recurrence in advanced high-grade neuroendocrine carcinoma (HGNEC) which included 53 LCNECs and 108 SCLCs (17); the LCNECs shared a worse overall response and survival outcomes (both PFS and OS) compared with SCLCs based on the PE regime. Besides, LCNECs are at high risk of brain recurrence when prophylactic cranial irradiation (PCI) is lacking. Most of the studies focused on advanced LCNEC. A recent study involved 56 patients for adjuvant chemotherapy, and SCLC-based regimens might be more effective than NSCLC-like therapy (P > 0.05), while no details such as tumor stage distribution and drugs usage were provided (10). We presented that in resected LCNEC, the PE regime might be a better choice for these patients and even acted as an independent prognostic factor for OS. While stage I patients were distributed more in the PE group (57.1%), the conclusion should be confirmed in the future. As genetic classification was implemented in clinical decision, some researches also explored genomic profiling in LCNEC. Zhuo et al. used next generation sequencing (NGS) to classify LCNEC as SCLC-like and NSCLC-like LCNEC (12), and treatment could be recommended based on genomic subtyping. Zhou et al. also provided the genomic landscape for LCNEC and indicated a group of gene alternations contained RUNX1, ERBB4, BRCA1, and EPHA3 (18), which may distinguish LCNEC from SCLC. Since the sample size was small (14 LCNECs and 10 SCLCs), more work should be undertaken in future investigations. Gene-based subtyping and further treatment options might emerge.

There are several limitations of the present study. First, the sample size was relatively small, and some data bias/missing may exist in a retrospective study; for instance, serum tumor markers were not detected in some patients for some unknown reasons, and we could not provide the missing part in present study, and the single-center samples with only Chinese ethnicity population involved in the present study may impede the capacity of obtaining robust conclusions to general populations; multicenter investigation in the future could be performed. Second, only resected patients were collected, and no advanced tumors were involved, then overview of LCNEC with different stages was difficult. Third, genomic and immune biomarkers such as PD-L1 information were insufficient. Since immunotherapy might be a choice in the future for LCNEC (19, 20), related immune markers should be investigated better.

In conclusion, LCNEC was a rare type of lung cancer with a high relapse rate. Our results demonstrated common driven gene mutants were scarce in LCNEC. Nodal (N) stage was associated with tumor recurrence and proved to be an independent prognostic factor for DFS, while OS significantly differed from different T stages. Serum positive CEA before surgery could be used for survival evaluation; besides, different adjuvant chemotherapies influenced the outcomes, with PE seemed a better choice. Perspective clinical trials were essential to provide a more confirmed conclusion, and deeper investigations of genomic/immune biomarker in LCNEC were also important.

## Data Availability Statement

The original contributions presented in the study are included in the article/**Supplementary Material**. Further inquiries can be directed to the corresponding author.

## Ethics Statement

The studies involving human participants were reviewed and approved by the Institutional Review Board (IRB) in ShangHai Chest Hospital [No. KS(Y)1982]. The patients/participants provided their written informed consent to participate in this study.

## Author Contributions

Conceptualization of the study was achieved by YS and BH. The research methodology was designed by YS, JX, and CL. Formal analysis of the data was conducted by YS, FH, and TC. Project administration was carried out by BH and XZ. The study resources were obtained by YS, JX and RZ. Software analysis of data and figures was conducted by YS and FH. In addition, supervision of the research was conducted by TC, XZ and BH. Writing the manuscript was carried out by YS and BH. Review and editing of the manuscript were carried out by TC and XZ. All authors contributed to the article and approved the submitted version.

## Funding

This work was funded by the “Star of SJTU” plan Medical-Engineering cross fund of Shanghai Jiao Tong University (YG2019QNA48). The funders had no role in the design and conduct of the study; collection, management, analysis, and interpretation of the data; preparation, review, or approval of the manuscript; and decision to submit the manuscript for publication.

## Conflict of Interest

The authors declare that the research was conducted in the absence of any commercial or financial relationships that could be construed as a potential conflict of interest.

## Supplementary Material

Supplementary Figure 1 Over-all survival (OS) for surgical resected LCNEC with different adjuvant chemotherapy strategies.
