# Supplementary figures and images for: Clinical Features and Outcomes Analysis of Surgical Resected Pulmonary Large-Cell Neuroendocrine Carcinoma With Adjuvant Chemotherapy
Source: Front Oncol. 2020 Dec 1;10:556194. doi: 10.3389/fonc.2020.556194 (PMC7736707; doi:10.3389/fonc.2020.556194)

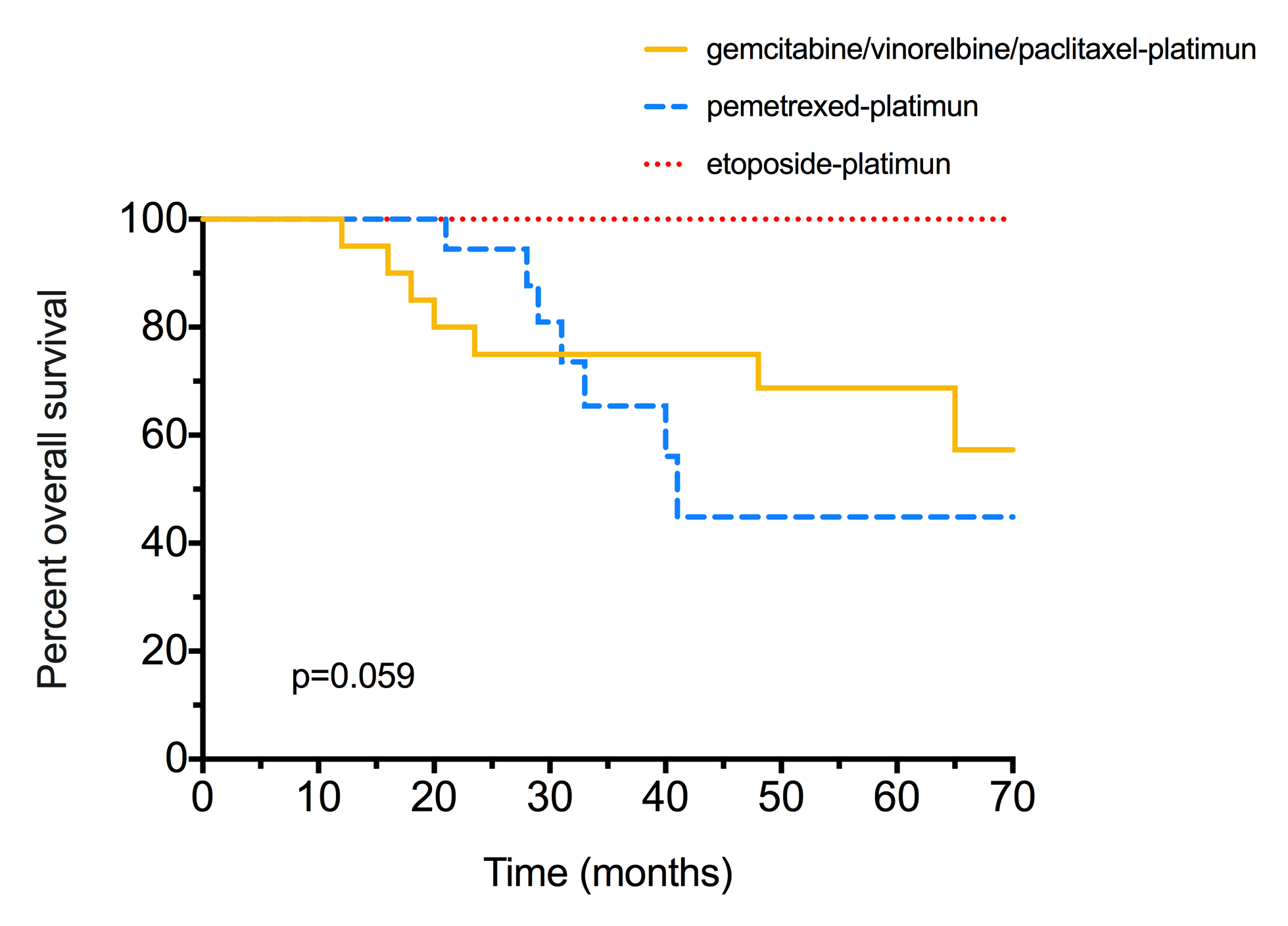

Supplement: Supplementary Figure 1 — Over-all survival (OS) for surgical resected LCNEC with different adjuvant chemotherapy strategies. [file Image_1.tiff]
